# ePath: an online database towards comprehensive essential gene annotation for prokaryotes

**DOI:** 10.1038/s41598-019-49098-w

**Published:** 2019-09-10

**Authors:** Xiangzhen Kong, Bin Zhu, Victoria N. Stone, Xiuchun Ge, Fadi E. El-Rami, Huangfu Donghai, Ping Xu

**Affiliations:** 1 Philips Institute for Oral Health Research, Virginia Commonwealth University, Richmond, Virginia 23298 United States of America; 2 Application Services, Virginia Commonwealth University, Richmond, Virginia United States of America; 3 Department of Microbiology and Immunology, Virginia Commonwealth University, Richmond, Virginia United States of America; 4 Center for Biological Data Science, Virginia Commonwealth University, Richmond, Virginia United States of America

**Keywords:** Genetic markers, Bacterial genes

## Abstract

Experimental techniques for identification of essential genes (EGs) in prokaryotes are usually expensive, time-consuming and sometimes unrealistic. Emerging *in silico* methods provide alternative methods for EG prediction, but often possess limitations including heavy computational requirements and lack of biological explanation. Here we propose a new computational algorithm for EG prediction in prokaryotes with an online database (ePath) for quick access to the EG prediction results of over 4,000 prokaryotes (https://www.pubapps.vcu.edu/epath/). In ePath, gene essentiality is linked to biological functions annotated by KEGG Ortholog (KO). Two new scoring systems, namely, E_score and P_score, are proposed for each KO as the EG evaluation criteria. E_score represents appearance and essentiality of a given KO in existing experimental results of gene essentiality, while P_score denotes gene essentiality based on the principle that a gene is essential if it plays a role in genetic information processing, cell envelope maintenance or energy production. The new EG prediction algorithm shows prediction accuracy ranging from 75% to 91% based on validation from five new experimental studies on EG identification. Our overall goal with ePath is to provide a comprehensive and reliable reference for gene essentiality annotation, facilitating the study of those prokaryotes without experimentally derived gene essentiality information.

## Introduction

Essential genes (EGs) are defined as those genes that are critical for the survival of an organism^1,2^. Identification and prediction of EGs are therefore of great importance for understanding cellular functions^3^, developing drugs against emerging pathogens and antibiotic-resistant pathogens^4,5^, and exploring evolutionary divergence^6^ as well as the origin of life^7^.

However, experimental identification of EGs in prokaryotes is costly and time-consuming^8^. Thus far, sufficient information on gene essentiality is only available for limited prokaryotic strains with genome-wide experimental data^9,10^ and the number is slowly increasing^11–13^. Many prokaryotic species are uncultivable, are too dangerous to handle, or have no genetic system available, making the experimental approach for EG identification unrealistic. Furthermore, available experimental results are derived from different methods in different instances and are more reliable for model organisms such as *Escherichia coli* and *Bacillus subtilis*. Generating these outcomes for other organisms is not a simple task.

*In silico* EG prediction emerges as a potential alternative method, which may greatly reduce cost in terms of both time and expense^14^. Computational methods for EG prediction are rapidly being developed, such as those using biological features of genes^15,16^, flux balance analysis of metabolic networks using constraint-based modelling^17^ and homolog and evolutionary distance^18^ combined with machine learning algorithms (e.g. support vector machine and artificial neural network (ANN))^18–20^. However, existing computational methods for EG prediction have several limitations. Predictions using metabolic models are constrained by the availability of the models corresponding to the organism of interest^21^. Moreover, these predictions are only available for those genes involved in metabolic pathways, whereas other genes such as those involved in genetic information processing and some cell envelope maintenance genes are excluded. In addition, computational methods using machine learning algorithms for EG prediction require existing gene essentiality information derived from laboratory experiments^18^ and extensive computational resources. Although they may show relatively high predictive power within their training sets, the general application of these tools remains largely uncertain outside their data domain. Moreover, these purely data-driven methods tend to establish quantitatively algorithms for EG prediction as a ‘black box’ (such as ANN), so that biological explanations underlying these methods are unclear. Overall, new methods for EG prediction with sound biological mechanisms and fast procedures, as well as databases for easy access to the prediction results, are highly desired for both genetic research and biological application.

**Figure 1:**
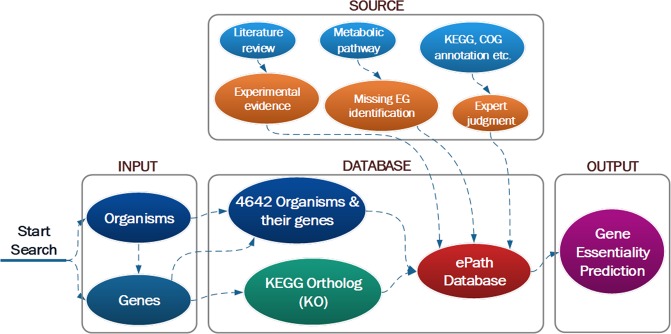
Conceptual diagram of the ePath online database and search engine.

We report here the development of the ePath database (Fig. 1) for EG annotation and prediction in prokaryotic genomes, covering the complete genomes of over 4,000 prokaryotic strains available in NCBI. We have proposed two criteria for EG prediction:

1. Genes serving the same molecular function but without any paralogs (isozyme or alternative pathway) should be consistently considered as either essential or non-essential.
2. A non-essential gene should be categorized as ‘genes playing essential functions but with paralogs (isozyme or alternative pathway) in the corresponding genome’, if there are EGs linked to both nodes of the corresponding edge in a KEGG pathway.

Accordingly, in ePath, the essentiality of genes is annotated based on two pieces of information. The first piece is the gene function annotation obtained from various databases. For a single gene, we retrieve the corresponding KEGG Ortholog (KO), and link the KO to a group of annotations including KEGG KO annotation, KEGG pathway/Module/Reaction annotation, Gene Ontology (GO), and Clusters of Orthologous Groups (COGs). We subsequently score the essentiality of this gene (‘P_score’ hereafter) based on the principle that a gene should be essential if it performs one of the following functions: genetic information processing, cell envelope maintenance or energy production^8^. The second piece is gene essentiality based on data provided from existing genome-wide experimental results. We have collected data from 31 strains listed in Database of Essential Genes (DEG)^9^ and linked all the experimental EGs to KO when possible, and summarized the essentiality frequency of these KOs in the 31 strains. Furthermore, we identify all the genes in the 31 strains if their projections on the KEGG metabolic pathway map (ko01100) have experimentally-verified EG neighbors on both sides of the edges, and consider these genes as ‘gap’ EGs that are missing in experiments (the ‘remapping’ algorithm). This is based on our previous finding using single gene-knockout technology that genes playing essential functions become non-essential when isozymes (paralogs or alternative pathway) exist in the genome^8^. For each specific gene, we thus develop an essentiality scoring criteria based on the essentiality of its orthologue in the 31 strains (E_score hereafter). Finally, the essentiality of this gene is annotated based on the E_score and P_score. The predictions are subsequently validated by five recent experimental studies that are not included in our training dataset. Overall, with the ePath database, we aim to provide a comprehensive and reliable reference for gene essentiality annotation with an easily accessible online database and searching tool, in order to facilitate studies for organisms lacking gene essentiality information. The ePath database is freely available at: https://www.pubapps.vcu.edu/epath/.

## Results

### Comparison of predicted and experimental EGs and the missing EGs

**Figure 2:**
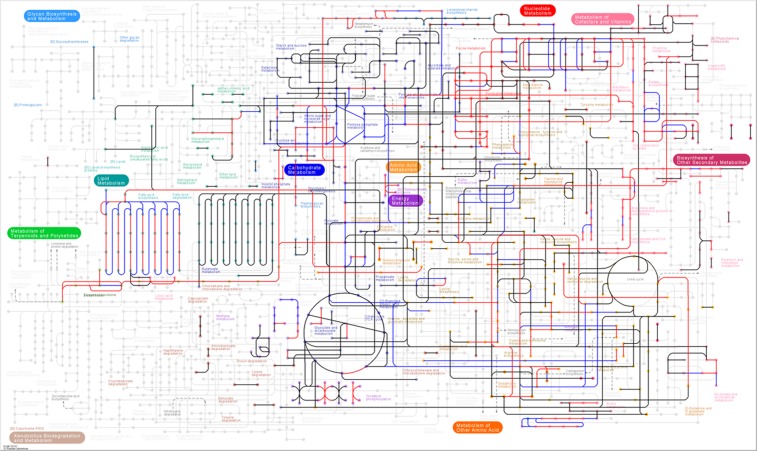
Metabolic pathway diagram of *E*. *coli* (eco01100 in KEGG pathway database^34^) with the gene essentiality information. The edges in red represent the EGs identified by experiment^22^. The edges in blue represent the missing EGs identified by the ‘remapping’ algorithm in this study. The edges in black represent the non-EGs. The original metabolic pathway map from KEGG^34^ is used with KEGG copyright permission number 190185.

We selected 31 strains in the DEG database with corresponding EGs identified experimentally (Table 1). These EGs were linked to KO numbers (see Table 2 for basic information). The E_score of every KO number was based on the knowledge of essential genes in these 31 strains. Predicted EGs were compared to the experimentally defined EGs and the missing EGs identified by the ‘remapping’ algorithm were highlighted. Among the 31 strains, 27 were found to possess missing EGs via the ‘remapping’ algorithm. The number of missing EGs ranged from 224 to 839 with an average value of approximately 350. Using *E*. *coli K12* as an example, we found that 3,139 genes in the genome were assigned with KO numbers. Experimentally, 296 genes were identified as essential^22^, among which 286 EGs were labeled on the KEGG pathway (eco01100) with KO numbers (Fig. 2). Our ‘remapping’ analysis showed another 469 genes were potentially essential that could have been missing in the experimental investigation.

**Table 1:** List of the 31 strains collected from the database of essential genes (DEG) used for training data and 5 strains collected from the literature used for validation.

No	Organism	KEGG abbreviation	EG number	Condition	Reference
***Training datasets***					
1	*Acinetobacter baumannii* ATCC 17978	acb	458	Rich medium	^35^
2	*Acinetobacter baylyi* ADP1	aci	499	Rich medium	^36^
3	*Agrobacterium fabrum* str. C58	atu	361	Rich medium	^37^
4	*Bacillus subtilis* 168	bsu	271	Rich medium	^1^
5	*Bacteroides fragilis* 638 R	bfg	547	Rich medium.	^38^
6	*Brevundimonas subvibrioides* ATCC 15264	bsb	412	Rich medium	^37^
7	*Burkholderia pseudomallei* K96243	bps	505	Rich medium	^39^
8	*Burkholderia thailandensis* E264	bte	406	Rich medium.	^40^
9	*Campylobacter jejuni* NCTC 11168 = ATCC 700819	cje	228	Rich medium	^41^
10	*Caulobacter crescentus*	ccr	480	Rich medium	^42^
11	*Escherichia coli* MG1655 II	eco	296	Rich medium	^22^
12	*Francisella novicida* U112	ftn	392	Rich medium	^43^
13	*Haemophilus influenzae* Rd KW20	hin	642	Rich medium	^44^
14	*Helicobacter pylori* 26695	hpy	323	Rich medium	^45^
15	*Mycobacterium tuberculosis* H37Rv III	mtu	687	Rich medium	^46^
16	*Mycoplasma genitalium* G37	mge	381	Rich medium	^7^
17	*Mycoplasma pulmonis* UAB CTIP	mpu	310	Rich medium	^47^
18	*Porphyromonas gingivalis* ATCC 33277	pgn	463	Rich medium	^48^
19	*Pseudomonas aeruginosa* PAO1	pae	336	Rich medium	^49^
20	CGA009	rpa	522	Rich medium	^50^
21	*Salmonella enterica* serovar Typhi Ty2	stt	358	Rich medium	^51^
22	*Salmonella enterica* serovar Typhimurium SL1344	sey	353	Rich medium	^51^
23	*Salmonella typhimurium* LT2	stm	230	Rich medium	^52^
24	*Shewanella oneidensis* MR-1	son	403	Rich medium	^53^
25	*Sphingomonas wittichii* RW1	swi	535	Rich medium	^54^
26	*Staphylococcus aureus* NCTC 8325	sao	351	Rich medium	^55^
27	*Streptococcus agalactiae* A909	sak	317	Rich medium	^56^
28	*Streptococcus pyogenes* NZ131	soz	241	Todd-Hewitt medium	^57^
29	*Streptococcus sanguinis* SK36	ssa	218	Rich medium	^8^
30	*Synechococcus elongatus* PCC 7942	syf	682	Rich medium	^58^
31	*Vibrio cholerae* N16961	vch	779	Rich medium	^59^
***Validation datasets***					
1	*Campylobacter jejuni* NCTC 11168	cje	166	Rich medium	^12^
2	*Mycobacterium tuberculosis* H37Rv	mtu	461	Rich medium	^11^
3	*Burkholderia cenocepacia* H111	bceo	398	Rich medium	^60^
4	*Herbaspirillum seropedicae* SmR1	hse	397	Rich medium	^61^
5	*Bacillus subtilis* 168	bsu	257	Rich medium	^13^

**Table 2:** Information for EGs collected from the database of essential genes (DEG).

Item	Value	Note
DEG strains included	31	—
Total genes	134,525	—
Essential genes	16,308	—
Essential genes with KO available	13,370	81.98%; 2,311 KOs without duplication
Non-essential genes	118,217	—
Non-essential genes with KO available	64,107	54.23%; 6,290 KOs without duplication
Total KO without duplication	6,839	31.10% of total 21,987 KOs in KEGG
Additional KO linked by #R	312	7,151 appear in at least one of the 31 strains
Total KO in database	21,987	—

We propose that the ‘gap genes’ identified by the ‘remapping’ algorithm from earlier experimental studies (Table 1) are non-EGs but playing essential functions, which could be largely attributed to the existence of isozymes, paralogs or alternative pathways in the genome. In this case, single gene-knockout technology cannot distinguish these ‘gap genes’ from the real EGs. In our previous work, we selected three pairs of paralogous or isozyme genes, SSA_0791/SSA_1494, SSA_0578/SSA_2195, and SSA_0352/SSA_1188, in *Streptococcus sanguinis* SK36. Indeed, double gene deletion mutants could not be constructed for these gene pairs, which supported our hypothesis^8^. Our ‘remapping’ algorithm therefore provides a new method to collect a comprehensive essential functions/reactions pool for EG prediction in prokaryotes. Overall, our approach in ePath is the annotation of ‘essential functions’ rather than ‘essential genes’, which is an important distinction for gene classification in prokaryotes^23^.

### KO essentiality scoring

**Figure 3:**
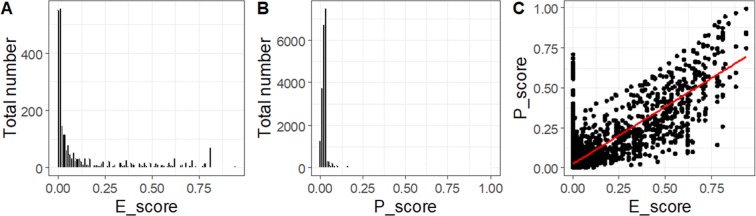
Frequency score distribution (**A**) E_score (only values higher than 0 are shown; N = 2546); (**B**) P_score (only values higher than 0 are shown; N = 21667); (**C**) correlation between P_score and E_score. The solid red line represents the best linear fit to the data with R^2^ = 0.67 (p < 0.001).

To expand the prediction for strains without experimental data, we attempted to calculate E_score and P_score for all 21,987 KOs. Note that 6,839 KOs (31.1%) appeared in at least one of the 31 strains, while the other KOs do not appear in any of the 31 strains. With 312 additional KOs that are linked using the reaction number in KEGG (#R), there are 7,151 KOs in total that can be assigned an E_score. Therefore, E_score was only available for 32.5% of the total KOs. For P_score, on the other hand, all 21,987 KOs were assigned. Analysis shows that distributions of E_score and P_score are both skewed to the left near zero (Fig. 3). E_scores range from 0 to 0.938, with an average value of 0.018 and standard deviation of 0.096. P_scores range from 0 to 0.997, with an average value of 0.037 and standard deviation of 0.084. We define the threshold for E_score as 0.6 and P_score as 0.03, which are both in the upper 90^th^ percentile of all the data. We note a significant positive correlation between E_score and P_score (R^2^ = 0.67, p < 0.001; Fig. 3), suggesting that these two criteria are closely related for EG prediction. As both E_score and P_score are readily available for KOs, essentiality of genes among the 4,642 strains can be evaluated as long as they have been assigned one KO number.

### ePath: the online EG database and search engine

**Figure 4:**
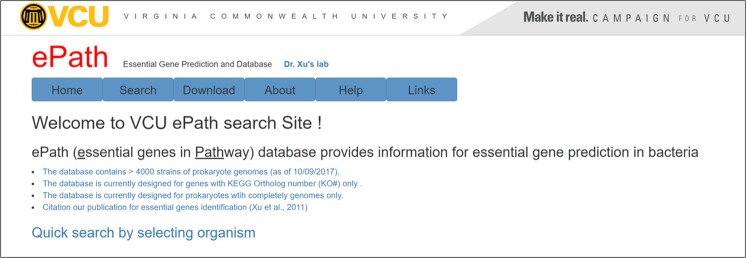
Interface of ePath website. The ePath searchable online database for essential genes for 4,000 + strains of prokaryote genomes.

Serving as an online EG database and search engine, the ePath website provides access to the information described above (Fig. 4). End users can access the data easily and freely. Different searching strategies are possible, including (1) search by organism name (e.g. Escherichia coli K-12 MG1655), (2) search by organism then by gene locus (e.g. Escherichia coli K-12 MG1655 then eco:b0002), (3) search by organism then by KO# (e.g. Escherichia coli K-12 MG1655 then K02313), and (4) search by organism then by gene name (Escherichia coli K-12 MG1655 then *purL*). The outcomes include Organism, KEGG abbreviation, Gene_Locus, KO number (KO_Nbr), Gene_Name, Gene_Function, E_Score and P_Score. For convenience, search results can be downloaded as a ‘.csv’ document. The entire database in ePath is also available for downloading and reanalysis by end users.

### Validation using new data

For validation, we collected five new datasets recently published for experimentally-derived EG identification, which did not include any of the 31 strains used for the training set (Table 1). The genes in these five datasets were used to query the ePath database for predictions (i.e. among the 4,642 strains). We compared the experimental results provided in each individual study with the EG prediction scores (E_score and P_score) from ePath, focusing on those genes with available KO numbers. Five criteria were applied for assessment of performance: (1) sensitivity; (2) specificity; (3) precision; (4) accuracy; and (5) F-measure (Table 3). Results showed that the proportion of essential genes that have been correctly identified ranged from 46% to 83% (sensitivity). Our method displayed better performance in predicting non-EGs (specificity) than EGs (precision), which ranged from 77% to 92% and 28% to 60%, respectively. The proportion of overall samples that were correctly identified (accuracy) ranged from 75% to 91%. The F-measure parameter indicates that harmonic mean of precision and sensitivity ranged from 34% to 70%. Our prediction performance was comparable to other approaches using either machine learning^24^ or evolutionary information^18^. Overall, the validation results indicate that our predictions are reliable and can serve as critical information for EG identification.

**Table 3:** Results for EG prediction validation.

Criterion	Calculation	cje	mtu	bceo	hse	bsu
sensitivity	TP/(TP + FN)	0.65	0.49	0.46	0.49	0.83
specificity	TN/(TN + FP)	0.77	0.87	0.89	0.87	0.92
precision	TP/(TP + FP)	0.31	0.60	0.28	0.32	0.60
accuracy	(TP + TN)/(TP + TN + FP + FN)	0.75	0.76	0.85	0.83	0.91
F-measure	2 × sensitivity × precision/(sensitivity + precision)	0.42	0.54	0.34	0.39	0.70

Note: (1) species abbreviations refer to Table 1. (2) **TP**: numbers of true positive; **FN**: numbers of false negative; **TN**: numbers of true negative; **FP**: numbers of false positive.

## Discussion

Genome-wide experimental efforts can be expensive and time-consuming, which has resulted in an increase in predictive methodologies. We present a quick and efficient tool to identify putative essential genes in prokaryotic species lacking genome-wide experimental data. The new tool (ePath) covers the completed genomes of over 4,000 prokaryotic strains, which is broader than approaches using metabolic models. ePath provides information to drive the study of essential genes. For example, (1) to understand important knockout genes due to paralogs^8^, (2) for elucidating gene functions of hypothetical genes (unpublished data), (3) For many organisms, experiments with Tn-seq and other whole-genome mutagenesis are difficult and time consuming. Their EG results are often difficult to assess. ePath predictions can provide independent information to evaluate these experimental datasets and assess the success of the mutagenesis methods used, and (4) to identify antibacterial targets for drug development^25,26^.

### Comparison with existing EG databases

Existing databases for EG information and annotations are generally for the currently available experimental outcomes. For example, the database of essential genes (DEG)^9^ contains collected and updated published experimental data concerning essential genes in different genomes. The Online GEne Essentiality database (OGEE)^10^, on the other hand, makes a step forward by collecting not only experimental outcomes but also gene features, so that there are possibilities to explore the distinction of EGs from non-EGs. Both the DEG and OGEE databases include comprehensive experimental essential gene data. Other studies attempt to either project experimentally identified EGs to functional roles in metabolic pathways^27^, or linking features of prokaryotic genes (e.g. genomic islands) to the possibility of a gene to be essential^25^. These studies significantly improve our ability and confidence in EG prediction in prokaryotes, but have not provided EG predictions of unknown organisms and their evaluations in these organisms.

The ePath database distinguishes itself from the other essential gene prediction resources by the following three distinct criteria: (1) end users of ePath may have access to EG annotations for over 4,000 prokaryotes with complete genome annotation available. This number is significantly higher than any other resource, potentially leading to more users and applications. (2) ePath demonstrates prediction accuracy ranging from 75%–91% based on validation. Comparatively, this performance is equivalent to other methods with similar objectives. However, in ePath, all prediction results are readily available so there is no requirement for further computation. The bias and uncertainty found in complicated machine learning are not present, making ePath more stable and comparable in EG predictions; and (3) ePath predicts EGs based on both sound principles from biological knowledge and existing experimental outcomes. The prediction algorithm in ePath is simple, facilitating its generalization to the whole prokaryote domain.

### Limitations and future perspectives

One of the limitations of ePath is that the EG predictions are only available for those genes with KO numbers available, which means that genes without functional annotation by KEGG (in most cases ‘hypothetical protein’) cannot be assessed. For example, there are 819 out of 2270 genes in *Streptococcus sanguinis* SK36 that have been annotated with KO. ePath can therefore provide predictions of essentiality to these 819 genes but not to the others. Despite the fact that ePath is limited by KO annotation, this limitation is continuously decreasing in importance, as the KEGG database performs updates on a regular basis, so that the number of genes with KO numbers available is rapidly increasing. We obtained KO numbers for the genomes of the 4,642 strains directly from the KEGG-KO database (as of October, 2017), and ePath will be updated following updates of the KEGG database in the future on a regular basis. Moreover, it appears that only a minor fraction of these ‘hypothetical genes’ without KO annotations are essential. For example, only 3 out of the 218 EGs in *Streptococcus sanguinis* SK36 are ‘hypothetical’^8^ and all of them are functionally related with the three basic categories (unpublished data). Meanwhile, KEGG also helpfully provides an online tool (BlastKOALA) for automatic KO assignment^28^. In theory, we can run BlastKOALA for all the 4,642 strains with available complete genome sequence data, which however would be computationally overwhelming. Therefore, we propose that the KEGG-KO database should be applied with caution for any of the annotated strains in KEGG. We suggest that for genes of interest to researchers but without KEGG annotations, it is possible to assign KOs using BlastKOALA. E_score and P_score could be obtained for the KOs using the KO essentiality by annotation table of the ePath website. With the accumulation of experimental data for essential genes in different organisms, more missed KO will be assigned with more accurate E_scores (and derived P_scores).

Another limitation that could be resolved in future studies is the determination of thresholds for the new E_score and P_score. To ascertain thresholds for newly proposed indicators, a large training dataset is usually required, which is difficult for EG studies. Although we observed good prediction performance with the E_score and P_score with validation data, determination of the thresholds for these two scores are based on quantitative decisions that require further evaluation. Given the small but increasing number of experimental EG studies, refinement of the scoring system for EG prediction in the near future is promising.

P_score is based on the assumption that one gene is more likely to be essential when it performs the functions among the three categories: cell envelope, energy production, and processing of genetic information, which, however, could be blurred as more refined functions may be found^23^. Nevertheless, P_score may nevertheless provide hints for gene essentiality. Therefore, we advocate that when ePath is used, E_score should be more weighted, while the P_score should be considered as supplementary information for EG prediction, especially when E_score is not available.

Finally, knowledge of paralogous genes would be beneficial for use of ePath. Notably, all EGs from DEG are identified using single gene knock-outs. Because of the existence of paralogous genes in prokaryotic species (isozymes or alternative pathways)^8^, even if a function is essential, the deletion of one paralogous gene may not lead to prokaryotic death (due to alternative gene functional compensation). In another case, if an essential compound is supplied in the growth condition (e.g. essential amino acid), the genes for related biosynthetic processes would not be essential under the experimental condition^8^. To resolve the issue, a remapping process is proposed in the present study to obtain a comprehensive essential function pool for the prediction of EGs in various species under different environmental conditions. However, this strategy brings some problems. In the case of a very high E_score indicating essentiality of a gene, a false positive prediction could be given due to the existence of paralogous genes or essential compounds, which results in low accuracy of EG predictions. End users could partially avoid these problems based on their knowledge of paralogous genes, isozymes and alternative pathways in the target species or the nutritional composition of their chosen growth medium.

## Materials and Methods

### EG annotation based on information of KO

As the first step, we collected all the KOs in the KEGG database (http://www.kegg.jp/kegg/) and their annotations from the database for gene annotation. There are 21,987 KOs ranged from “K00001” to “K21987” in KEGG (as of October, 2017). For each KO, we collected its annotation from the KO database (http://www.kegg.jp/kegg-bin/get_htext). These KO annotations are molecular-level functions determined from experimental evidence of functionally characterized sequence data. They are positioned as nodes in networks and are defined in the context of KEGG molecular networks (KEGG pathway maps, BRITE hierarchies and KEGG modules)^29,30^. Among all 21,987 KOs, 665 do not have KO annotations. These KOs were therefore marked as “0” for further analysis. In addition, we collected information for the KOs including gene names and descriptions given by RefSeq^31^ or GenBank^32^, as well as the corresponding KEGG pathway (#*ko*), KEGG module (#*M*) and KEGG Reaction (#*R*) from KEGG Brite Database (http://www.kegg.jp/kegg/brite.html)^33^. In particular, each *#ko* is composed of three layers of annotation, e.g., for *#ko00010*, the pathway annotation is “Metabolism −>Carbohydrate metabolism −>Glycolysis/Gluconeogenesis”. Note that for one certain KO, there can be more than one (or none) corresponding #*ko*, #*M* or #*R*. We assigned COGs and GOs number to each KO according to the “binary relationships” provided by KEGG Brite Database, which also could be more than one or none. For COGs, their categories and annotations were collected from NCBI (ftp://ftp.ncbi.nih.gov/pub/COG/COG2014/static/lists/listCOGs.html). Furthermore, GO annotations were collected from the Gene Ontology Consortium (http://geneontology.org/page/download-annotations), in particular the “UniProt [multispecies], no IEA annotations”. Overall, we have collected multiple functional annotations for each of the 21,987 KOs. We therefore have established the first database for this study, which is presented in detail on the ePath online database.

### EG annotation based on existing experimental discovery

We collected a group of strains (Table 1) from the database of essential genes (DEG: http://www.essentialgene.org/)^9^, for which EGs have been identified and validated using experimental approaches. The selected 31 strains used as the training set include 134,525 genes in total, in which 16,308 genes were identified as EG using experimental methods, mostly in rich media (Table 2). For all 134,525 genes, we assigned KOs to each by implementing the BlastKOALA tool (http://www.kegg.jp/kegg/tool/annotate_sequence.html), which determines the most appropriate KO for one gene based on a modified version of the KOALA algorithm after the BLAST search against a non-redundant dataset of pan-genome sequences generated from the KEGG GENES database^28^. If one gene is annotated with more than one KO, we selected the best match provided by BlastKOALA. As the input to the BlastKOALA, the genome sequences for the 31 strains were collected from NCBI together with the ‘gene_id’, which is also provided by the DEG. This variable therefore serves as the linkage between the outcomes from BlastKOALA and the DEG. All 31 strains have been included in KEGG so that the GENES family/genus abbreviation (Table 1) was pre-assigned after uploading the genome sequence in BlastKOALA. Among the 16,308 EGs, 13,370 were successfully linked to one KO (2,311 KOs without duplication), while for the rest of the 118,217 non-EGs, 64,107 had a KO available (6,290 KOs without duplication). Therefore, 77,477 KOs were obtained in total, belonging to 6,839 KOs after the removal of duplicates. The details of the gene information above are presented in the ePath online database. We also elaborated to match each of the 6,839 KOs with other KOs if they share the same *#R*, as we hope to add additional information to understand KO function. This procedure resulted in an additional 312 KOs. These 7,151 KOs were further analyzed in the following sections. The details of the gene information above are presented in the ePath online database.

### Remapping: a new algorithm for identifying missing EGs

We used KEGG pathways to identify those potential EGs that could have been overlooked in experiments with a new algorithm called ‘remapping’. As the first step, we collected the ‘Locus-tag’ for all the EGs from DEG, which is critical for subsequent analysis with KEGG pathways labeled by the ‘Locus-tag’ along the edges of the pathway map. Due to a recent update of NCBI, the ‘Locus-tags’ for many strains have been changed. We therefore obtained the ‘Locus-tag’ for all the EGs in the 31 strains from their original publications (Table 1). We assigned the ‘Locus-tag’ for all the 16,308 EGs (except for 102 missing).

Next, we focused on the metabolic pathways in the category of “Global and overview” maps in KEGG, i.e., the ‘ko01100’ pathway. We downloaded this pathway file in “.xml” format from KEGG for all the 31 strains, using the python package ‘requests’. The pathway document contains the information for all the chemical reactions of the metabolism with the corresponding functional genes in the organism according to state-of-the-art knowledge in literature^29^. The nodes serve as chemical compounds either as substrate or products, while all the edges act as the KO group(s) that produce the enzymes for the reaction. For any given strain, the edges are labeled by the corresponding genes’ ‘Locus-tag’.

For each strain, we further parsed the “.xml” file for the ‘ko01100’ pathway using the python package ‘xml.etree.ElementTree’. By collecting the attributions with ‘entry’ and type ==’gene’, we obtained the gene ‘Locus-tag’ list and the corresponding reaction ID list within the pathway. In addition, the ‘reaction’ attribution provides the reaction information including the compound id for the substrate and product. Based on the table for the linkage between #*R* and #*K*, we further determined all the corresponding KO for each reaction in the pathway. Overall, we summarized all the information from the pathway’s.xml file. We established a table for each strain accordingly, in which each row represents a biochemical reaction and the columns were as follows: substrate(s) (#*C*), product(s) (#*C*), reactions (#*R*), KOs (#*K*), and gene ‘Locus-tag’. Furthermore, for each reaction, we queried the corresponding genes to the EGs database above based on ‘Locus-tag’. We scored the reaction with 5 points if at least one gene was essential, and scored the reaction with 1 point if all the genes were non-essential. We therefore built a chemical reaction matrix (*S*) for all the compounds in each strain, serving as the linkage matrix for one pathway. The element *S*_*ij*_ (*i* = 1, 2, …, *n*; *j* = 1, 2, …, *n*) represents the existence and essentiality of the reaction between two compounds, where *i* and *j* are the location of the element representing the chemical’s index, and *n* is the total number of chemicals. For essential genes, *S*_*ij*_ = 5; for non-essential genes, *S*_*ij*_ = 1; and for reactions that do not exist, *S*_*ij*_ = 0. With the *S*_*ij*_ available for one strain, we then went through the KEGG pathways and identified the missing EGs that were not experimentally determined. We used the Depth-first search (DFS) algorithm combined with our criteria for traversing the pathway map represented by the matrix *S*. We started the search from each row in the matrix representing one edge in the map. The algorithm for DFS can be described as follows:

- The search will go across the next edge either if the edge represents one EG, or if there is at least one EG linked to the node on the other end of the edge.
- The search will stop if the edge represents one non-EG and there is no EG linked to the node on the other end of the edge.

**Figure 5:**
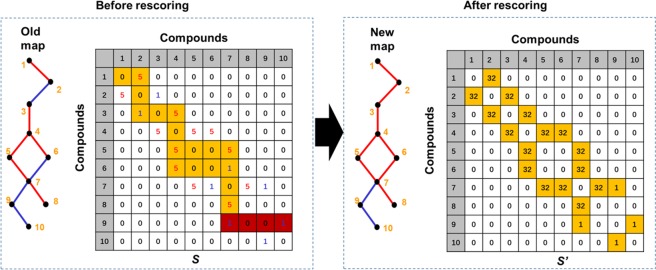
Link missing essential genes in pathway. An illustrative example of the ‘remapping’ algorithm processed on the KEGG pathway map with 10 hypothetical compounds. The left panel represents the map and matrix (*S*) before the rescoring. In the old map (left panel), the blue edges are non-essential genes, while the red ones are essential genes. The elements in the matrix (*S*) show the existence and essentiality of the reaction between the two corresponding compounds. The colored elements highlight how the DFS algorithm searches for the linked edges for the first edge. The yellow boxes are the linked edges and the red boxes are the discarded edges. The right panel shows the new map and the updated matrix (*S’*). Note that in the new map, edges (2–3) and (6–7) are considered as the missing EGs and are labeled red. The *S’* provides the final score for each edge, which serves as the basis for EG determination.

After DFS, we would be able to identify multiple ‘essential sections’ inside the pathway, in which all the missing EGs would be included and identified. Then, we rescored the starting edge by summing all the scores of the edges linked to the starting edge identified by the DFS search, and we obtained the updated matrix (*S’*). Note that in the KEGG pathways, one gene may appear in multiple edges in one map. If one non-essential gene is identified as a missing EG at one location, all the other edges where this gene appears will be labeled as essential, as will all the other genes in those edges. This situation could produce an infinite loop. Therefore, we only ran the search once for the sake of simplicity. We provided an illustrative example for the algorithm with 10 chemicals (genes) in Fig. 5.

### EG scoring system

Two dimensions of scoring for KO essentiality are developed based on the data collected. First, an experimental score (E_score) is assigned for each of 7,151 KOs. E_score is based on the appearance and essentiality of each KO among the 31 stains. We propose a formula for calculating E_score_i_ for gene *i* (Eq. 1) in the range of 0–1, where a higher value suggests a higher potential for essentiality.

1 $${{E}}_{-}{{score}}_{i}={(\frac{E{G}_{i,e}+E{G}_{i,m}}{E{G}_{i,e}+E{G}_{i,m}+nonE{G}_{i}+1})}^{2}\times (\frac{E{G}_{i,e}+E{G}_{i,m}+nonE{G}_{i}}{31})$$

where *EG*_*i*,*e*_ represents the number of strains that have a particular KO that is essential according to experimental outcomes; *EG*_*i*,*m*_ represents the number of strains that have a particular KO that is missing essential according to ‘remapping’; *nonEG*_*i*_ represents the number of strains that have a particularKO that is not essential. The first term on the right of Eq. 1 represents the probability of the KO as EG among the strains in which it appears. The second term on the right of Eq. 1, on the other hand, indicates the probability of the KO to appear among strains as another aspect of the gene’s essentiality.

Second, a prediction score (P_score) was assigned for each KO. The P_score originates from the E_score and was determined by expert judgment on the essentiality of the KO based on comprehensive annotations for prokaryotes. For a KO without an E_score, at first two additional scores, i.e. P_score_KEGG and P_score_COG, were determined based on the KEGG or COG annotation and calculated from the E_score of the KOs with the same pathway annotation. These two scores were manually assigned within a range of 0–1. For example, K15792 has no E-score, but its KEGG pathway annotation is “Lysine biosynthesis//Peptidoglycan biosynthesis//”. There is one KO (K01928) with E_score 0.59 for the same KEGG pathway annotation. As a result, P_score_KEGG for K15792 is 0.59. Its COG annotation is “UDP-N-acetylmuramyl tripeptide synthase//UDP-N-acetylmuramyl pentapeptide synthase//”. However, no KO with the same COGs and with available E_score is found. Therefore, P_score_COG for K15792 is 0. As another example, K10781 has no E-score, but its KEGG pathway annotation is “Fatty acid metabolism//Fatty acid biosynthesis//”. There are 8 other KOs with the same annotation (K01716, K18473, K00645, K02371, K00648, K10780, K00667, and K00668) with an average E_score of 0.57. Therefore, P_score_KEGG of K10781 is 0.57. There is no COG annotation. There are a total of 1292 empty cells for COG annotations with average E_score 0.04. As a result, P_score_COG of K10781 is 0.04. The rationale originates from the earlier findings^8^ that KOs belonging to the following three functional categories are essential: (1) gene information processing, (2) energy production, and (3) cell envelope. We manually examined all KO annotations with E_score and found that the KOs with higher E_scores were highly related with the three functional categories above. To expand the prediction for KOs without E_scores, P_score is assigned as the average of standardized P_score_KEGG and P_score_COG for each KO. Note that we cannot assign P_score for the KOs having neither KEGG pathways nor COG annotations. Overall, a P_score may range from 0 to 1, with 0 representing no potential for essentiality, while 1 denotes certain essentiality based on expert judgment.

### Essential gene prediction for 4,642 strains

We collected all the prokaryotes in KEGG Organisms database (in total 4,642, as of October, 2017). For the genomes of each collected strain, we assigned a KO number based on the existing information in KEGG (KEGG-KO database). For all the 4,642 strains, KO annotations for genes in each genome were collected by parsing the ‘.keg’ file via python, which serves as the basis for further EG annotation based on both E_ score and P_score. All EG prediction results for the 4,642 strains are provided in the ePath online database.
